# Effects of Different SARS-CoV-2 Testing Strategies in the Emergency Department on Length of Stay and Clinical Outcomes: A Randomised Controlled Trial

**DOI:** 10.1155/2024/9571236

**Published:** 2024-02-14

**Authors:** Kira Louisa Boldt, Myrto Bolanaki, Fabian Holert, Antje Fischer-Rosinský, Anna Slagman, Martin Möckel

**Affiliations:** Charité-Universitätsmedizin Berlin, Berlin, Germany

## Abstract

The turn-around-time (TAT) of diagnostic and screening measures such as testing for SARS-CoV-2 can affect a patient's length of stay (LOS) in the hospital as well as the emergency department (ED). This, in turn, can affect clinical outcomes. Therefore, a reliable and time-efficient SARS-CoV-2 testing strategy is necessary, especially in the ED. In this randomised controlled trial, *n* = 598 ED patients presenting to one of three university hospital EDs in Berlin, Germany, and needing hospitalisation were randomly assigned to two intervention groups and one control group. Accordingly, different SARS-CoV-2 testing strategies were implemented: rapid antigen and point-of-care (POC) reverse transcription polymerase chain reaction (rtPCR) testing with the Roche cobas® Liat® (LIAT) (group one *n* = 198), POC rtPCR testing with the LIAT (group two *n* = 197), and central laboratory rtPCR testing (group three, control group *n* = 203). The median LOS in the hospital as an inpatient across the groups was 7 days. Patients' LOS in the ED of more than seven hours did not differ significantly, and furthermore, no significant differences were observed regarding clinical outcomes such as intensive care unit stay or death. The rapid and POC test strategies had a significantly (*p* < 0.01) shorter median TAT (group one 00:48 h, group two 00:21 h) than the regular central laboratory rtPCR test (group three 06:26 h). However, fast SARS-CoV-2 testing strategies did not reduce ED or inpatient LOS significantly in less urgent ED admissions. Testing strategies should be adjusted to the current circumstances including crowding, SARS-CoV-2 incidences, and patient cohort. This trial is registered with DRKS00023117.

## 1. Introduction

Since the World Health Organisation declared the SARS-CoV-2 pandemic on 11 March 2020 [1], the impact of the virus has strained healthcare systems worldwide [2]. One reason for this has been the increase in inpatient length of stay (LOS). Compared to prepandemic times, the median LOS in German hospitals rose in 2020, despite lower hospital occupancy rates [3].

Prolonged hospitalisation is known to increase complications and mortality [4, 5], as well as financial inefficiency [6]. Therefore, reducing inpatient LOS is considered of major importance. Early detection of other viral infections, such as influenza, via point-of-care (POC) testing has proven to shorten the time until specific treatment was started [7] and to promote timely transfer to specialised wards in order to reduce inpatient LOS [8].

Emergency departments (EDs) function as a central hub for patients entering the hospital and have a strong influence on intraclinical processes, particularly by providing early diagnostics, such as screening for infectious diseases.

Personnel in EDs are commonly confronted with a great workload especially due to high patient volumes and crowding. While discussing the reasons for crowding, at least three different influences should be addressed. Asplin et al. fittingly call them input, throughput, and output [9].

Some input factors are difficult to solve from within the emergency department. For example, the increased proportion of low-acuity attendances [10] or frequent presenters [11] aggravated by systemic problems related to poor coordination between different ambulatory health care facilities needs to be addressed on a larger scale. In addition, the individual use of EDs depending on their location and the urbanisation of the surrounding area must be taken into consideration [12].

In terms of throughput, EDs must quickly adapt to new circumstances. During the COVID-19 pandemic, for example, all ED patients with an indication for hospital admission got tested for SARS-CoV-2 [13] regardless of their symptoms. This procedure seems self-evident, as patients infected with SARS-CoV-2 can present clinically unapparent or nonspecific while being highly infectious [14]. Thus, laboratory diagnostics are a necessary screening tool to prevent nosocomial infections [15].

Concerning the ED LOS specifically, Sangal et al. observed an increase in the time patients spent in the ED after this screening policy was implemented [16]. Research has shown associations between longer ED LOS and an increased risk of SARS-CoV-2 transmission [17, 18], as well as worse clinical outcomes [19, 20].

So, while early identification of SARS-CoV-2 infected patients in the ED is of utmost importance, particularly with a view to the patient's subsequent stay in the hospital, it simultaneously seems to slow down treatment and therefore the ED's throughput. Waiting for a screening result, and thus deciding whether a patient needs to be transferred to a specialised ward and/or special isolation measures are necessary, influences the output point mentioned by Asplin et al. For this reason, analysing and optimising testing procedures may be a central approach to reducing the LOS in EDs.

The gold standard for detecting SARS-CoV-2 infections is the real-time polymerase chain reaction (rtPCR) test [13]. Early on in the pandemic, POC diagnostic instruments became available, such as the Roche cobas® Liat® (LIAT), a POC rtPCR test for SARS-CoV-2 with high sensitivity and specificity [21]. Rapid antigen tests were also quickly developed and implemented; however, they are not solely suitable to rule out SARS-CoV-2 infections due to their reduced sensitivity [22]. Still, they have the potential to reduce ED LOS, as presented by Bond et al. [23].

There are numerous studies evaluating POC rtPCR approaches for SARS-CoV-2 testing, mostly underlining their reliability and potential to reduce time to results when compared to standard laboratory rtPCR [24]. However, no sufficient data exist for the impact of SARS-CoV-2 POC rtPCR compared to other test strategies on patient care after initial ED evaluation. Therefore, the aim of the randomised controlled trial presented here was to determine if different testing strategies in the ED influence LOS and clinical outcomes of patients who are admitted to the hospital.

## 2. Materials and Methods

### 2.1. Trial Design and Participants

**Figure 1:**
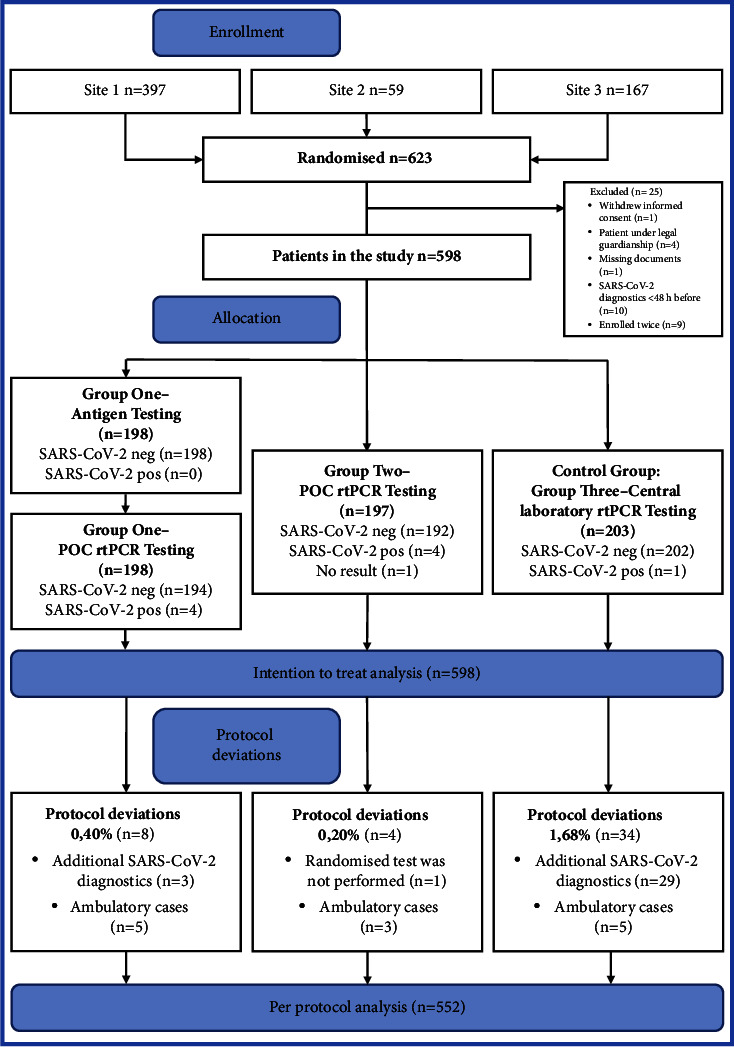
Trial design and participant flow. Note: flow diagram of the progress through the trial and analyses.

The current study is a multicenter, open-label, randomised controlled trial. The study was conducted at Charité–Universitätsmedizin Berlin, which is a maximum care facility at level three and one of the largest university hospitals in Europe [25]. Patients were enrolled from three separate EDs, all of which belong to the Charité–Universitätsmedizin Berlin: “Internal Medicine ED Campus Virchow-Klinikum” as site 1, “Surgical Medicine ED Campus Virchow-Klinikum” as site 2, and “Central ED Campus Charité Mitte” as site 3. Recruitment ran from 5 May to 28 September 2021, and follow-up continued until the end of each participant's inpatient treatment. Patients were evenly randomised into two intervention groups and one control group according to the SARS-CoV-2 testing strategies in the ED (Figure 1). The trial ended after the estimated sample size was achieved; data collection and statistical analyses were performed in the following seven months.

Since this study seeks to evaluate SARS-CoV-2 screening instruments, all adult ED patients with the need for inpatient admission, who were willing and able to give written informed consent, were eligible for study participation. Patients requiring urgent intervention after ED admission received direct POC testing to prevent time delay as presented by Möckel et al. [21] and were therefore excluded from this study. Patients who were younger than 18 years, under legal guardianship, and those who had received SARS-CoV-2 rtPCR testing within the last 48 hours or presented to the ED multiple times during study conduction were excluded too. Eligibility criteria did not change after trial commencement.

### 2.2. Intervention

**Figure 2:**
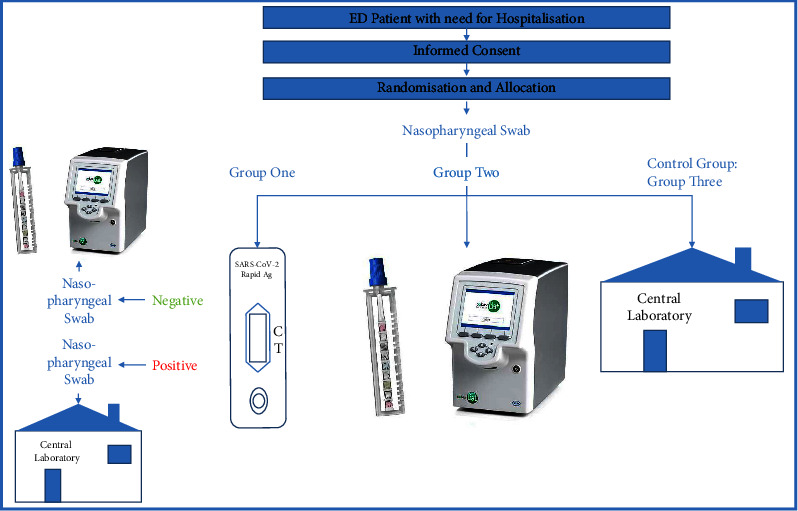
Study design. Note: visualisation of the study design.

Eligible patients were approached by study personnel or attending physicians and randomly allocated to one of three groups with different SARS-CoV-2 diagnostic strategies after giving written informed consent. Group one was assigned to rapid antigen and subsequent rtPCR testing. These involved two nasopharyngeal swabs: one for the Roche® SARS-CoV-2 rapid antigen test, and in case of a negative or invalid result, a second swab was analysed with the LIAT. A positive antigen test result would have been confirmed with an rtPCR performed in the central laboratory for semiquantification. The second group exclusively received a POC rtPCR test with the LIAT. Trained ED personnel performed all rapid antigen and LIAT tests at a suitable workstation within the department. The third group's swabs were directly sent to the central laboratory for rtPCR testing (Figure 2). The results of all antigen tests were manually transferred into the patients' digital file, while all LIAT and central laboratory systems were already digitally integrated, allowing for direct access to results.

### 2.3. Outcomes

The primary outcome was inpatient LOS following ED presentation in days. Secondary outcomes were ED LOS and the turn-around-time (TAT) for SARS-CoV-2 testing, measured from first sampling until final result delivery. Outcomes and measures were not changed after the trial commenced.

Information on the satisfaction of patients was documented using the validated ZUF-8 Score [26]. All other data were retrospectively collected from the hospital information system and added to a REDCap® based online survey by study personnel.

### 2.4. Sample Size

The primary study hypothesis was a shorter inpatient LOS when comparing groups one or two to group three. With alpha = 0.05 and an aspired power of 80%, a study size of at least *n* = 143 per group was determined. The equivalence of hospital LOS for groups one and two was assumed. To define the sample size, the alpha error was adjusted to 0.025 which sets the required participants to a minimum of *n* = 173 per group for equivalence tests with a three-day distribution in LOS.

Because of expected drop-outs and cross-overs, a uniform group size of at least *n* = 200, and therefore, a total number of *n* = 600 cases was aimed for.

### 2.5. Randomisation and Masking

A researcher with no further clinical involvement in the study created a random allocation sequence using an online tool (“Simple Randomiser Tool” by Sealed Envelope Ltd. [27]) for block randomisation with block sizes of 12. Subsequently, the group allocation documents were sealed inside opaque envelopes according to the sequence numbers. After a participant had signed informed consent, the documents were opened by a study nurse or attending physician, and the intervention was started accordingly.

### 2.6. Statistical Methods

Data are expressed as absolute and relative frequencies or median and interquartile ranges (IQR). Baseline characteristics and outcomes were compared between groups with a Kruskal–Wallis test. *p* values <0.05 (two-sided) are considered statistically significant. Bonferroni corrected *p* values are provided for all confirmatory analyses. Statistical analysis was performed using IBM SPSS version 28 software for Microsoft Windows. The primary analysis was an intention-to-treat (ITT) analysis. In addition, the results of a per-protocol (PP) analysis were reported.

To evaluate the patients' satisfaction with their ED stay, the average sum of all completed ZUF-8 questionnaires was calculated, as recommended by Schmidt et al. [26].

### 2.7. Ethical Statement

The study was performed in line with the principals of the Declaration of Helsinki and in accordance with Good Clinical Practice Guidelines. The Institutional Ethical Review Board of the Charité–Universitätsmedizin Berlin granted ethical approval (12 February 2021, processing number EA4/027/21).

## 3. Results

### 3.1. Participant Flow and Study Population

Figure 1 demonstrates the participant flow through the trial and analysis period. 598 participants constituted the ITT analysis: group one *n* = 198, group two *n* = 197, and group three *n* = 203. In the PP analysis, *n* = 552 patients were included, divided into group one *n* = 190, group two *n* = 193, and group three *n* = 169. Two cases were lost to follow-up because medical records for their treatment following ED presentation could not be obtained.

Patients' characteristics and baseline data were comparable across the randomised groups. 44.80% of the patients in the study population identified themselves as female. *N* = 444 individuals (76.90%) had been vaccinated against SARS-CoV-2 at least once prior to inclusion. Information on which vaccine they received was provided by *n* = 408 patients, as shown in Table 1.

### 3.2. Outcomes and Estimation

In the ITT and PP population, the median LOS for hospitalisation after the ED visit was seven days for every group (*p* = 0.86) (Table 2). The varying SARS-CoV-2 testing strategies resulted in different TATs. Table 2 presents these significantly different time intervals (*p* < 0.01).

All SARS-CoV-2 tests were medially started more than four hours after a patient's arrival in the ED (Table 2). Furthermore, no significant differences were detected between the patients' allocation to a normal ward, ICU care, or outpatient treatment (ambulatory case) after ED stay and hospitalisation (Table 2). 338 patients fully completed the ZUF-8 questionnaire. Out of the maximum attainable test score of 32 points, the median satisfaction rate was 29 points (Table 2).

Following the primary ED presentation, 42 of 596 patients had to be admitted to an ICU, and 20 of 596 persons died during hospitalisation. The occurrence of these events and the median days spent in the ICU are also shown in Table 2.

20 of the 598 patients in the study died during the inpatient stay (Table 2). Statistically, no significant association with the randomised testing strategy could be found (*p*=0.766). The median time of death was 19 days after admission (IQR 9.25–34). In one of the deceased individuals with a preexisting oncological disease, SARS-CoV-2 RNA was detected in the initial rtPCR (ct > 30). Based on a second rtPCR with a negative result, this was interpreted as residually positive: a condition that regularly occurs in immunocompromised patients [28]. Furthermore, the deceased patients' tests were medially started at 03:02 h after presentation in the ED (IQR 02:21 h–04:45 h), which is an hour earlier than compared to the total study population. The most common documented causes of death were acute cardiac arrest, respiratory failure, and sepsis. None of the patients were actively infected with SARS-CoV-2 during their inpatient stay. Thus, no association between the testing strategy and patient death could be established.

The analysis of the patients' clinical data showed that the study protocol was broken by attending physicians in 32 cases. While the rtPCR results within the study protocol were still pending, additional SARS-CoV-2 diagnostics were started. In 29 of the 32 cases (90.63%), the patients were originally allocated to the control group (central laboratory rtPCR) and received additional SARS-CoV-2 diagnostics in the ED. The additional measures consisted of rapid antigen testing (4/29; 13.79%), POC rtPCR (21/29; 72.41%), or fast rtPCR testing via the central laboratory (4/29; 13.79%), the latter also having a shorter TAT but not being part of this study's protocol. The responsible physicians were asked to give a reason for the protocol breaches, and all of them stated that a quicker test result was necessary to allow transfer to an inpatient department. LOS calculations were repeated as PP analyses to determine if the aforementioned protocol breaches altered any results (Table 2, Figure 1) without showing grave differences.

Inpatient main and secondary diagnoses were monitored and compared between the groups of the ITT population, as shown in Tables 3 and 4. Heart failure, atrial fibrillation and flutter, and diseases of the biliary tract were most frequently documented as main diagnoses in the ITT analysis. The most commonly documented secondary diagnoses included essential hypertension and metabolic disorders.

## 4. Discussion

The study compared the impacts of three different testing strategies for SARS-CoV-2 in a university ED setting: (1) rapid antigen testing + POC rtPCR testing, (2) POC rtPCR, and (3) central laboratory rtPCR. There was no observable impact of the SARS-CoV-2 testing strategy in the ED on the patients' ED and inpatient LOS in this population.

### 4.1. Interpretation

The same inpatient LOS and distribution of participants to subsequent wards, as well as the same clinical outcomes, show that different SARS-CoV-2 test regimens do not significantly affect the patients' outcomes in the further clinical course under these study conditions.

The equally nonsignificant difference in ED LOS suggests that a change in the SARS-CoV-2 testing strategy may not be the only influencing factor in shortening it.

Recent literature showed contradictory effects regarding patients diagnosed via POC or rapid testing in the ED. Some researchers have been successful in reducing the LOS in the ED using a POC testing approach, applied to all laboratory diagnostics [23] or especially concerning SARS-CoV-2 testing, as demonstrated by Baron et al. [29]. However, similar to the trial presented here, this strategy did not lead to the same results in other studies. Neither Hausfater et al. [30] nor Asha et al. [31] were able to achieve a significant reduction in ED LOS through POC testing. In other studies, specifically related to infectious respiratory diseases such as influenza, a reduction in ED LOS was again possible [7, 32].

From this and the results presented here, it can be concluded that POC diagnostics may offer advantages, but they have to be combined with the removal of further exit block obstacles to achieve a reduced ED LOS.

Given the negligible impact of the testing strategy in this trial, other determinants like availability of beds or required diagnostics during the ED stay are likely to be more important. It stands to reason that patients' ED LOS is dependent on several further external and internal factors. These particularly involve the target department in context with a patient's comorbidities and symptom complexity [33].

Another relevant influence on the ED LOS lies in the reduction of bed capacities due to the resource shift towards COVID-19 care at the time of study conduction, in that it might have delayed patient allocation after ED treatment was finished.

As anticipated, the TAT of both POC testing strategies was significantly shorter than the central laboratory analyses, taking the latter more than six hours medially until result transmission. Considering the patients' median LOS in the ED of seven hours and more, one could argue that conducting SARS-CoV-2 rtPCRs in the central laboratory allows for enough time to attain the screening result before planned patient transfer and not lead to holding periods after the finished ED assessment (Table 2). This approach, however, would require all tests to be started within the first hour after initial ED presentation during which the indication for hospital admission and, therefore, infectiological screening of patients without the need for immediate intervention might not be clear. Testing all ED patients regardless of their need for inpatient treatment could strain personnel-, material-, and laboratory capacities.

In addition, the TAT of rtPCRs conducted in the central laboratory varied profoundly, as seen in the IQR of 05:22 to 09:46 hours (Table 2). This could be due to different laboratory capacity utilisation as not only ED samples but also those from planned hospital admissions and in-house screenings are analysed. Overall, the exclusive use of central laboratory diagnostics appears to be less reliable in terms of timing and could therefore delay the planning of further diagnostics as well as increase the time to transfer if dependent on the SARS-CoV-2 test result. A combination with or exclusive use of POC tests could avoid these difficulties.

29 patients that were randomised to group three (rtPCR in the central laboratory, longest TAT) received additional SARS-CoV-2 diagnostics in the ED with shorter TATs to fasten their transfer. Otherwise, these patients would have had to remain in the ED until rtPCR results were available. This would have considerably prolonged the ED stay in the form of a so-called exit block. During the study, an overall tendency towards diagnostic approaches for SARS-CoV-2 with shorter TATs was noticed.

### 4.2. Trial Limitations and Generalisability

The relatively low SARS-CoV-2 incidence rates in Berlin (median of 48,06/100.000 [34]) and the predominant delta-variant during the data collection period are limitations of this study. In addition, more than three fourths of the study population were vaccinated against SARS-CoV-2. These factors, as well as seasonal effects on SARS-CoV-2 dynamics, may have further contributed to a very low occurrence of COVID-19 in the observed patients. Higher incidences and the dominance of more infectious SARS-CoV-2 subvariants, which show a tendency towards immune evasion (like it is the case for Omicron [35]), might have altered the results of this trial. Since the recruitment of this study was finished, more specific SARS-CoV-2 treatments (e.g., nirmatrelvir or specific antibody therapy) became available. If the patient cohort had consisted of more SARS-CoV-2-infected patients that had to be hospitalised for COVID-19, the reduced TAT of the LIAT might have allowed for earlier start of treatment and quicker disposition, reducing these patients' ED and inpatient LOS.

Since many parameters were retrospectively documented from clinical routine data, not all datasets are complete. Nevertheless, comparable numbers could be analysed for all allocation groups.

Although the patient recruitment took place in three separate EDs, the study sites are comparable because of similar organisational structures, a shared pool of available inpatient beds, and collectively offered medical services. Still, a selection bias, caused by the exclusive recruitment of study patients at Charité–Universitätsmedizin Berlin, should be taken into account.

Finally, it must be noted that all patients signed informed consent before being enrolled in the study. Therefore, patient groups under legal guardianship without sufficient German language skills or those with cognitive limitations are not represented here.

## 5. Conclusions

This randomised controlled trial showed that rapid or POC SARS-CoV-2 testing strategies do not significantly reduce LOS in the hospital or the ED in less urgent admissions when used as a screening instrument.

Nevertheless, the impact of factors improving ED-specific internal processes should be considered. With quicker SARS-CoV-2 test results, the number of isolation rooms and amount of personal protective equipment used could be reduced. If indicated, now established COVID-19-specific therapies could be started earlier, and staff and fellow patients could more easily be protected from nosocomial infections through cohort building. This continues to make POC diagnostics highly appealing for infectious diseases such as SARS-CoV-2.

In general, testing strategies should be adjusted to current circumstances: high influx, crowding situations, times with very high SARS-CoV-2 incidences, or when treating patients who are considered to suffer a potentially severe COVID-19 course and thus require quick testing and specific COVID-19 therapy, the reliable and time-efficient SARS-CoV-2 diagnostic via POC with a device such as the LIAT seems preferable.

## Figures and Tables

**Table 1:** Baseline data.

	Total *n* = 598	Group one-antigentest + point of care rtPCR test, *n* = 198	Group two-pointof care rtPCR test, *n* = 197	Control group: group three-centrallaboratory rtPCR test, *n* = 203	Total *n*(mis) =	*p* value (two-sided) =
Age (years)	65 (51–77)	63 (49–75)	65 (52–77)	67 (55–78)	0	0.054
Sex (female)	268 (44.80%)	83 (41.90%)	96 (48.70%)	89 (43.80%)	0	0.374
SARS-CoV-2 vaccinated	444 (76.90%)	144 (74.60%)	143 (75.30%)	157 (87.30%)	21	0.27
Vaccine					36	0.11
Comirnaty (BioNTech)	302 (74.02%)	102 (77.27%)	89 (68.46%)	111 (76.03%)		
Spikevax (Moderna)	51 (12.50%)	19 (14.39%)	14 (10.77%)	18 (12.33%)		
Vaxzevria (AstraZeneca)	28 (6.86%)	5 (3.79%)	13 (10%)	10 (6.85%)		
Janssen (Johnson & Johnson)	6 (1.47%)	0 (0%)	5 (3.85%)	1 (0.68%)		
Multiple vaccine types	21 (5.15%)	6 (4.55%)	9 (6.92%)	6 (4.11%)		
Risk factors						
Smoking	122 (20.40%)	36 (18.20%)	45 (22.80%)	41 (20.20%)	23	0.529
Obesity = BMI > 29.9	115 (19.40%)	42 (21.20%)	43 (21.80%)	30 (14.80%)	30	0.126
Vital parameters						
Systolic blood pressure (mmHg)	134 (120–149)	134 (122–150)	131.50 (121–146)	134 (118–150)	23	0.563
Diastolic blood pressure (mmHg)	80 (70–89)	81 (71–90)	80 (70–90)	80 (70–86.50)	23	0.174
Heart rate per minute	88 (76–101)	89 (78–102.25)	86 (76–102)	87 (74–99)	44	0.178
Body temperature (°C)	36.70 (36.50–37.10)	36.80 (36.50–37.10)	36.70 (36.50–37.20)	36.70 (36.40–37.10)	30	0.338
Respiratory rate per minute	16 (15–18)	16 (15–18)	16 (15–19)	16 (15–18)	196	0.588
Oxygen saturation (%)	99 (97–100)	99 (98–100)	98 (97–100)	99 (97–100)	26	**0.012**
Symptoms						
Fever	106 (17.70%)	36 (18.20%)	37 (18.80%)	33 (16.30%)	4	0.837
Dry cough	46 (7.70%)	20 (10.10%)	14 (7.10%)	12 (5.90%)	31	0.747
Haemoptysis	6 (10%)	3 (1.50%)	0 (0.00%)	3 (1.50%)	30	0.693
Sore throat	11 (1.80%)	0 (0.00%)	5 (2.50%)	6 (3.00%)	32	0.32
Rhinitis	7 (1.20%)	3 (1.50%)	1 (0.50%)	3 (1.50%)	32	0.584
Headache/melalgia/muscular pain	68 (11.40%)	16 (8.10%)	26 (13.20%)	26 (12.80%)	33	0.351
Dyspnea	156 (26.10%)	44 (22.20%)	57 (28.90%)	55 (27.10%)	30	0.324
Gastrointestinal problems	212 (35.50%)	73 (36.90%)	70 (35.50%)	69 (34.00%)	29	0.925
Charlson Comorbidity Index	2.00 (1.00–4.00)	2.00 (0.00–3.25)	2.00 (0.50–4.00)	2.00 (1.00–4.00)	0	0.704
Pregnancy (women only)	2 (0.30%)	0 (0.00%)	0 (0.00%)	2 (1.00%)	59	0.058
Laboratory values						
pH	7.39 (7.36–7.42)	7.39 (7.36–7.43)	7.39 (7.37–7.42)	7.39 (7.36–7.42)	38	0.391
Sodium (mg/dL)	139 (136–141)	139 (135–142)	139 (135.75–141)	139 (136–141)	30	0.978
Potassium (mg/dL)	4.10 (3.80–4.50)	4.10 (3.80–4.40)	4.10 (3.80–4.50)	4.10 (3.80–4.50)	24	0.61
Glucose (mg/dL)	122 (106–152)	122 (107–153)	121 (105–153)	122.5 (108.00–149.25)	33	0.675
Lactate (mg/dL)	14.00 (10.00–19.25)	14.00 (10.00–20.50)	14.00 (9.00–18.00)	14.00 (10.00–21.00)	40	0.183
CRP (mg/L)	20.20 (4.10–76.70)	21.80 (3.48–77.53)	20.15 (4.68–76.63)	19.00 (4.30–76.80)	105	0.968
Leucocytes (nL)	8.78 (6.59–11.85)	9.34 (7.30–12.44)	8.58 (6.48–11.79)	8.10 (6.35–11.39)	11	**0.048**
PCT (*μ*g/L)	0.15 (0.06–0.47)	0.18 (0.07–0.66)	0.16 (0.07–0.60)	0.13 (0.60−0.33)	350	0.228
SARS-CoV-2 test result					0	0.477
Negative	588 (98.30%)	194 (98.00%)	192 (97.50%)	202 (99.50%)		
No test	1 (0.20%)	0 (0.00%)	1 (0.50%)	0 (0.00%)		
Positive	9 (1.50%)	4 (2.00%)	4 (2.00%)	1 (0.50%)		
Positive with ct > 30	6 (66.67%)	4 (100%)	2 (50.00%)	0 (0.00%)		0.317
Positive with ct < 30	3 (33.33%)	0 (0.00%)	2 (50.00%)	1 (100%)		

*Note*. Baseline characteristics are shown for the total study population and each group. Median (IQR) or absolute frequency (relative frequency as valid percentage) is shown. Because not all datasets were complete, the number of missing values for each item is noted as “Total *n* (mis) =”. Positive SARS-CoV-2 results were divided into cycle threshold (ct) values > 30 and ct < 30. Bold values indicate that p values < 0.05 are significant.

**Table 2:** Outcomes.

	Total *n* = 598	Group one-antigentest + point of care rtPCR test, *n* = 198	Group two -point of care rtPCR test, *n* = 197	Control group: group three-centrallaboratory rtPCR test, *n* = 203	Total *n*(mis) =	*p* value (two-sided)=
Inpatient LOS						
Intention to treat analysis	7.00 d (4.00–12.00)	7.00 d (4.00–11.00)	7.00 d (4.00–12.75)	7.00 d (7.00–14.00)	2	0.86
Per protocol analysis	7 d (4.00–13.00)	7 d (5.00–11.00)	7 d (4.00–13.00)	7 d (4.00–14.00)	2	0.98
ED LOS						
Intention to treat analysis	07:38 h (05:51–10:21)	07:32 h (05:59–10:19)	07:37 h (05:50–09:36)	07:43 h (05:45–10:48)	0	0.96
Per protocol analysis	07:34 h (05:45–10:01)	07:32 h (05:59–10:19)	07:39 h (05:48–09:36)	07:25 h (05:26–10:11)	0	0.54
TAT						
Intention to treat analysis	00:48 h (00:21–05:27)	00:47 h (00:42–00:56)	00:21 h (00:21–00:21)	06:26 h (05:22–09:46)	5	**<0.01**
Time to teststart						
Intention to treat analysis	04:09 h (02:44–05:52)	04:10 h (02:37–05:26)	04:23 h (03:02–06:21)	03:47 h (02:39–05:50)	5	0.08
Clinical course after ED					2	0.88
Direct transfer to ICU	9 (1.51%)	3 (1.52%)	3 (1.52%)	3 (1.48%)		
Hospitalisation in Charité	505 (84.45%)	168 (84.84%)	164 (83.25%)	173 (85.22%)		
Hospitalisation in external clinics	51 (8.53%)	17 (8.59%)	19 (9.64%)	15 (7.39%)		
Ambulatory case	27 (4.52%)	9 (4.55%)	9 (4.57%)	9 (4.43%)		
Dismissal against medical advice	6 (1.00%)	1 (0.51%)	2 (1.02%)	3 (1.48%)		
Clinical course during hospitalisation						
ICU admission	42 (7.02%)	16 (8.08%)	12 (6.09%)	14 (6.90%)	4	0.75
LOS ICU	5.50 d (2–11.25)	4.50 d (2.00–9.75)	6.50 d (3.25–14.75)	5.50 d (2.75–12.25)	4	0.68
Clinical course after hospitalisation					2	0.97
Dismissal home	512 (85.91%)	166 (83.84%)	173 (88.27%)	173 (85.64%)		
Rehabilitation	37 (6.21%)	15 (7.58%)	11 (5.61%)	11 (6.45%)		
Death in hospital	20 (3.36%)	4 (2.02%)	7 (3.57%)	9 (4.46%)		
Transfer to another clinic	18 (3.02%)	8 (4.04)	4 (2.04%)	6 (2.97%)		
Nursing home	9 (1.51%)	5 (2.53%)	1(0.51%)	3 (1.49%)		
Satisfaction with ED treatment						
ZUF-8 score out of 32 points	29 (26–31)	29 (26–31)	28 (26–30)	29 (26–31)	260	0.9

*Note*. Inpatient length of stay (LOS) in days (d), emergency department (ED) LOS in hours (h), turn-around-time (TAT) of allocated test strategy in hours (h) measured from first sample taking to result transmission, clinical course after ED discharge, clinical course during hospitalisation, clinical course after hospitalisation, and satisfaction with ED treatment measured with ZUF-8 scores are shown for the total study population and each group. The median (IQR) or absolute frequency (relative frequency as valid percentage) is shown. Because not all datasets were complete, the number of missing values for each item is noted as “Total *n* (mis) *=*”. Bold values indicate that p values < 0.05 are significant.

**Table 3:** Most common main diagnoses.

ICD-10-code	Diagnosis	Number	Percent (%)
I50	Congestive heart failure	(19/228)	8.33
I48	Atrial fibrillation and flutter	(8/228)	3.51
D63	Anaemia in chronic diseases	(5/228)	2.19
A41	Sepsis	(5/228)	2.19
B96	Specified bacterial agents as the cause of diseases	(5/228)	2.19
C92	Myeloid leukaemia	(5/228)	2.19
J44	Chronic obstructive pulmonary disease	(5/228)	2.19
K80	Cholelithiasis	(5/228)	2.19
K85	Acute pancreatitis	(5/228)	2.19

*Note.* Of *n* *=* 228 different main diagnoses assigned, the most frequent are shown here classified in accordance to the ICD (International Statistical Classification of Diseases and Related Health Problems) system version 10.

**Table 4:** Most common secondary diagnoses.

ICD-10-code	Diagnosis	Number	Percent (%)
I10	Essential (primary) hypertension	(89/1698)	5.24
U99	Special screening examination for SARS-CoV-2	(87/1698)	5.12
Z11	Special screening examination for infectious and parasitic diseases	(87/1698)	5.12
E87	Disorders of fluid, electrolyte, and acid-base balance	(53/1698)	3.12
E78	Disorders of lipoprotein metabolism and other lipidaemias	(41/1698)	2.41
I25	Chronic ischaemic heart disease	(41/1698)	2.41
I50	Congestive heart failure	(41/1698)	2.41
E11	Type 2 diabetes mellitus	(36/1698)	2.12
B96	Specified bacterial agents as the cause of diseases	(25/1698)	1.47

*Note.* Of *n* *=* 1698 different secondary diagnoses assigned, the most frequent are shown here classified in accordance to the ICD (International Statistical Classification of Diseases and Related Health Problems) system version 10.

## Data Availability

The data used in this study have not been made available because of patient data protection. Anonymised data can be obtained on reasonable request from the corresponding author.

## Abbreviations

TAT: Turn-around-time
LOS: Length of stay
ED: Emergency department
POC: Point-of-care
rtPCR: Reverse transcription polymerase chain reaction
LIAT: Roche cobas® Liat®
IQR: Interquartile ranges
ITT: Intention-to-treat
PP: Per-protocol.
